# Induction and Prevention of Gastric Cancer with Combined *Helicobacter Pylori* and Capsaicin Administration and DFMO Treatment, Respectively

**DOI:** 10.3390/cancers12040816

**Published:** 2020-03-28

**Authors:** Faisal Aziz, Mingxia Xin, Yunfeng Gao, Abhijit Chakroborty, Imran Khan, Josh Monts, Kjersten Monson, Ann M. Bode, Zigang Dong

**Affiliations:** 1 The Hormel Institute, University of Minnesota, Austin, MN 55912, USA; faziz@umn.edu (F.A.);; 2 China–US (Henan) Cancer Institute, Zhengzhou, Henan 450003, China; 3 Department of Ophthalmology, Mayo Clinic, Rochester, MN 55902, USA

**Keywords:** capsaicin, DFMO, gastric inflammation, gastric cancer, *Helicobacter. pylori*, IL-6

## Abstract

Gastric cancer risk evolves over time due to environmental, dietary, and lifestyle changes, including *Helicobacter pylori* (*H. pylori*) infection and consumption of hot peppers (i.e., capsaicin). *H. pylori* infection promotes gastric mucosal injury in the early phase of capsaicin exposure. This relationship suggests a need to investigate the mechanism of how both *H. pylori* infection and capsaicin contribute to gastric inflammation and lead to gastric cancer. C57-Balb/c mice were infected with the *H. pylori* (SS1) strain and then fed capsaicin (0.05% or 0.2 g/kg/day) or not. Consequently, tumor size and phenotype were analyzed to determine the molecular mechanism driving the shift from gastritis to stomach cancer. Moreover, we used 2-difluoromethylornithine (DFMO) in mice to prevent gastric tumorigenesis by reducing inflammation and promoting recovery of disease-free stasis. This study provides evidence showing that a combination of *H. pylori* infection and capsaicin consumption leads to gastric carcinogenesis mediated through interleukin-6 (IL-6) stimulation with an incidence rate of 50%. The anti-inflammatory role of DFMO highlights the injurious effect of inflammation in gastric cancer development and the need to reduce gastric inflammation for cancer prevention by inhibiting IL-6. Accordingly, preventive measures such as reduced capsaicin consumption, *H. pylori* clearance, and DFMO treatment may lessen gastric cancer incidence.

## 1. Introduction

The Centers for Disease Control and Prevention estimates that roughly two-thirds of the population of the world harbor *Helicobacter pylori* (*H. pylori*), a bacterium that colonizes the stomach and duodenum [1,2]. Without treatment, *H. pylori* infection is a known risk factor for gastrointestinal illnesses like chronic gastritis, peptic ulcers, and stomach cancer [3,4,5]. In 2001, an epidemiologic study demonstrated that patients infected with *H. pylori* were nearly six times more likely to develop gastric cancer compared to uninfected people. Other gastric cancer risk factors include host [6,7], dietary, and lifestyle practices including capsaicin consumption, stress levels, and ingestion of inflammatory foods or beverages. In summary, the etiology of gastric cancer is influenced by bacterial variability, along with host genetic and environmental factors; however, the molecular mechanisms governing these factors have not been fully elucidated [8,9,10,11]. 

Evidence suggests that *H. pylori* infection impacts the development and progression of gastric mucosal injury during early capsaicin exposure from hot pepper consumption [12]. Capsaicin reportedly suppresses immune function and increases host susceptibility to infection [13,14,15]. Other data suggest an epidemiological link between stomach cancer incidence and chili pepper-rich diets [12,16]. In contrast, several studies reported the anti-cancer and anti-inflammatory effects of capsaicin that activate the expression of several genes involved in the inhibition of cancer cell survival, growth arrest, angiogenesis, and metastasis [17,18,19,20]. Ultimately, capsaicin’s role in carcinogenesis remains controversial. Additionally, synergism between *H. pylori* and other factors is not often the focus of studies. This neglected interplay between *H. pylori* infection and other agents may be critical to the initiation and persistence of gastric inflammation, as well as the development of gastric cancer. Here, we explore the combined effect of *H. pylori* infection and capsaicin exposure on the development of gastritis and gastric carcinogenesis, as well as possible anti-inflammatory regimens for disease prevention. The selected anti-inflammatory agent, DFMO (2-difluoromethylornithine), reportedly alters immune populations within the tumor microenvironment and inhibits tumor growth by increasing CD8^+^ infiltration [21,22]. Despite its chemopreventive potential, the anti-inflammatory and anti-cancer effects of DFMO against gastritis and gastric cancer are unknown. 

Additionally, the role of capsaicin consumption in the development of gastritis and gastric cancer has not been experimentally elucidated before now. Here, we explore the combined effect of *H. pylori* infection and capsaicin consumption on the development of gastritis and, later, gastric cancer. Lastly, we highlight DFMO as an anti-inflammatory treatment against gastritis and as a preventative measure against gastric carcinogenesis. 

## 2. Results

### 2.1. Capsaicin and H. pylori Infection Induce Gastritis Leading to Gastric Cancer

**Figure 1:**
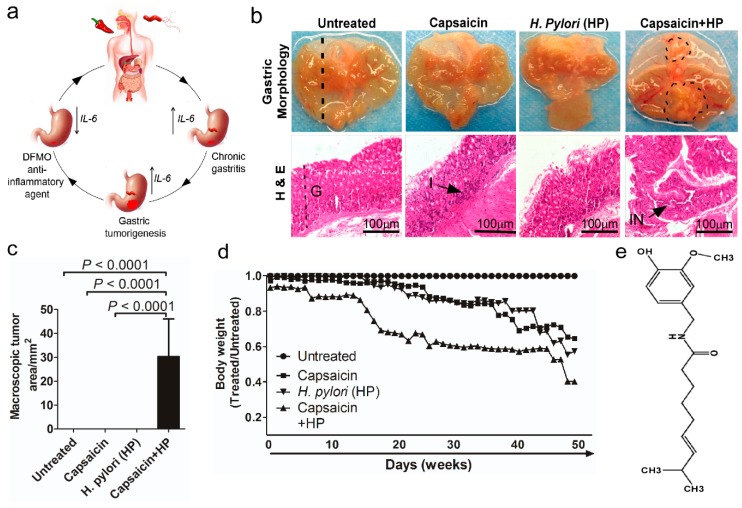
Development of mouse models for capsaicin- and *H. pylori*-induced progression of gastritis to gastric cancer. (**a**) Schematic drawing showing the effect of capsaicin consumption and *H. pylori* infection in the development and progression of gastritis to gastric tumorigenesis mediated through the stimulation of IL-6. (**b**) Macroscopic *(top)* and histologic *(bottom)* analyses of gastric mucosae (*n* = 5/cohort). Images of tumors at 52 weeks from mice infected with *H. pylori* and treated with capsaicin; tumor areas are outlined with dotted circles. Hematoxylin & eosin (H&E)-stained cross-sections are cut along the fundus to the proximal end of the corpus, as shown by the straight dotted line. Gastric pathology was substantially changed after 32 or 52 weeks of combination treatment with *H. pylori* and capsaicin. The panels are magnified 10× (bar, 100 μm) and 40× (bar, 20 μm). Arrow and G = glandular zone, I = inflammatory cells, IN = muscular mucosae invasion. (**c**) The size of gastric tumors from mice treated with capsaicin and infected with *H. pylori* compared to mice treated with only capsaicin or *H. pylori* is shown. (**d**) Effect of capsaicin, *H. pylori*, or a combination of capsaicin with *H. pylori* infection on mouse body weights. Body weights are shown as treated/untreated. (**e**) The chemical structural of capsaicin. Data are expressed as mean values ± SEM, (*n* = 5/cohort).

To investigate the role of capsaicin consumption in the progression of *H. pylori*-associated gastric cancer, we developed a mouse model treated with a combination of capsaicin and *H. pylori* infection-induced gastritis, leading to gastric cancer. Macroscopic morphometric analysis revealed that capsaicin consumption induced gastric inflammation (at 32 weeks) as the initiating process, which led to tumor development (at 52 weeks) in the stomachs of mice with *H. pylori* infection (Figure 1b). Tumor growth in mice treated with both capsaicin and *H. pylori* confirmed the role of a combination of capsaicin and *H. pylori* in gastric tumorigenesis. Macroscopic analysis of tumor area revealed the development of tumors only in mice treated with both *H. pylori* and capsaicin (30.26 ± 7.017 mm^2^; *p* < 0.0001; Figure 1c). Mice treated with a combination of *H. pylori* and capsaicin showed significantly lower body weight compared to untreated mice (Figure 1d). Overall, these results indicated that *H. pylori* and capsaicin together caused a progressive shift from gastritis to gastric cancer. These results provided experimental animal evidence showing the combined effect of capsaicin and *H. pylori* infection on the development of gastric cancer. Histological analysis also showed multifocal elongation of the gastric pits, glandular atrophy, and a significant reduction in the glandular zone in atrophic foci. These changes were significantly less in mice treated with capsaicin and infected with *H. pylori* exhibiting tumors (0.2 ± 0.2; *p* < 0.0001), compared to mice treated with only capsaicin (2.60 ± 0.24; *p* = 0.02) or infected with *H. pylori* only (1.0 ± 0.316; *p* = 0.0002; Figure S1a–d). 

### 2.2. Mice Treated with Capsaicin and H. pylori Exhibited Increased Gastric Atrophy and Accelerated Tumor Growth

**Figure 2:**
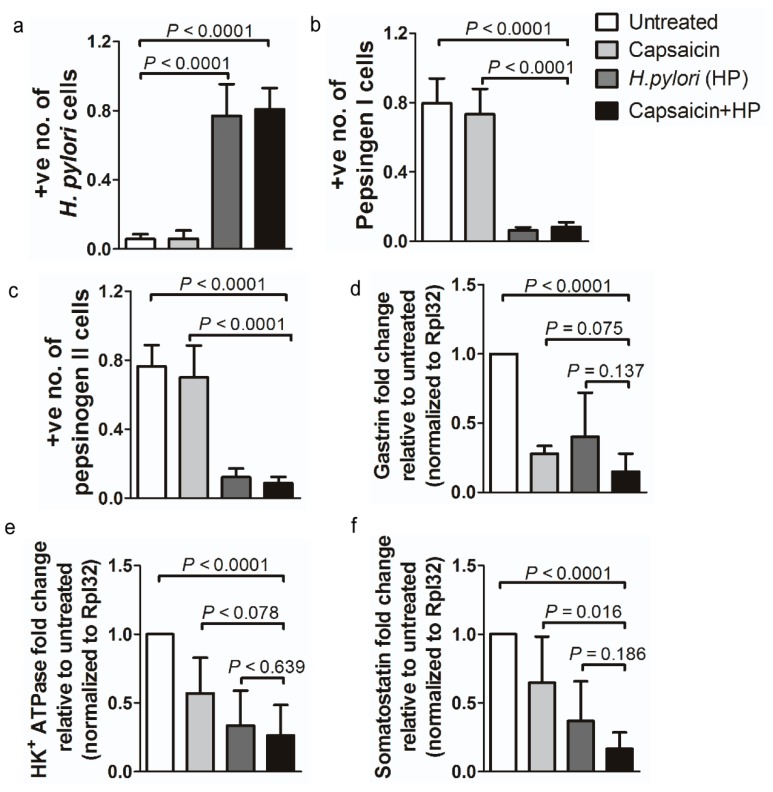
Immunohistochemical and qPCR analysis of gastric disease mouse models. Immunohistochemical detection of pepsinogen I and II and *H. pylori* expression in gastric tissues from mice treated with both capsaicin and *H. pylori*. Paraffin-embedded gastric tissues were stained to detect *H. pylori*, pepsinogen I, or pepsinogen II, respectively. Quantitative immunohistochemical detection of (**a**) *H. pylori*, (**b**) pepsinogen I, or (**c**) pepsinogen II expression. A strong intensity was observed in untreated gastritis tissue compared to gastric cancer tissues. Scale bars represent 100 μm (10×), and the scale bar in the inset images represents 20 μm (40×). A score of 1 denotes the highest staining, whereas a score of 0 indicates negative staining. mRNA analysis of (**d**) *gastrin*, (**e**) *H^+^K^+^ ATPase*, and (**f**) *somatostatin* by qPCR. Average fold change in mRNA relative to untreated control (*n* = 5/cohort).

Histologic analysis revealed significant glandular atrophy in the fundi of mice at 32 weeks of a combination treatment with capsaicin and *H. pylori* compared with untreated mice or mice treated only with capsaicin or *H. pylori* (Figure 2). Dual treatment resulted in a reduction of parietal cell components, especially parietal and chief cells, which were replaced by gastric mucus-producing cells. Pepsinogen I and II, gastrin, somatostatin (gastric endocrine regulators), and H+ /K+ ATPase (parietal and chief cells) showed substantially lowered expression in mice exhibiting gastric tumorigenesis compared with untreated mice or mice treated singularly (0.264 ± 0.09; *p* < 0.0001; Figure 2a–f). Gastrin, somatostatin, and H+/K+ ATPase are required for normal gastric mucosal development and parietal cell activation [23,24]. In mice exhibiting carcinogenesis, these biomolecules were almost completely ablated. As a result, a combination of capsaicin and *H. pylori* directly accelerated loss of differentiated epithelial cell types, leading to chronic atrophic gastritis and eventually to gastric tumorigenesis. The combination treatment shifted gastric tissue to a tumor phenotype with increased numbers of inflammatory cells found in the tumor gastric mucosa. This observation suggests that *H. pylori* or capsaicin alone is not enough to cause gastric tumorigenesis and that a combination is required to trigger the formation of submucosal glands and invasion of tumor cells. 

### 2.3. Diminished Expression of Tumor Suppressor Genes Coincided with Accelerated Tumorigenesis 

The effect of a combination of capsaicin and *H. pylori* was associated with increased tumor progression accompanied by substantially reduced expression of several gastric tumor suppressor genes (TSGs), including *Tff1* by *qPCR* (0.273 ± 0.120), *Tff2* (0.126 ± 0.078), *Gkn1* (0.143 ± 0.045), and *Gkn2* (0.172 ± 0.060) in the group of mice that developed tumors compared to other groups (*p* < 0.0001; Figure S2a–d). Loss of these gastric-specific TSGs promotes tumorigenesis [25,26,27]. *Tff1* and *Tff2* genes are upstream regulators of *gastrokine (Gkn)* gene expression. The GKN2 protein exists as a heterodimer with *Tff1*, and the *Tff2* genes are upstream regulators of *gastrokine (Gkn)* gene expression. Therefore, loss of *Tff1 or Tff2* expression (TFFs peptides) could lead to tumor growth [28]. Overall, inhibition of *Tff1*, *Tff2*, *Gkn1*, and *Gkn2* is known to be associated with tumorigenesis [28,29]. Gkns are gastric tissue-specific Tsgs, which are affected by *H. pylori*, cytokines, and Tff. Gkns are highly expressed in normal gastric tissues and downregulated in gastric cancer. Overexpression of Gkn2 could be associated with the prognosis of gastric cancer patients, as shown to inhibit gastric cancer cell proliferation, migration, and invasion [30,31,32]. TFF peptides (TFF1, TFF2, and TFF3) are involved in several steps of gastric cancer development through multiple oncogenic pathways and are considered to be potential tumor suppressor genes, including *PI3-kinase*, *phospholipase C (PLC)*, *MAPK*, *PKC*, and the rapamycin target *mTOR*, but also *EGF-R*, *COX-1*, *COX-2,* and G-protein coupled receptors [33,34,35,36].

### 2.4. Mice Administered Capsaicin and H. pylori Displayed Increased Gastric Tissue Damage

**Figure 3:**
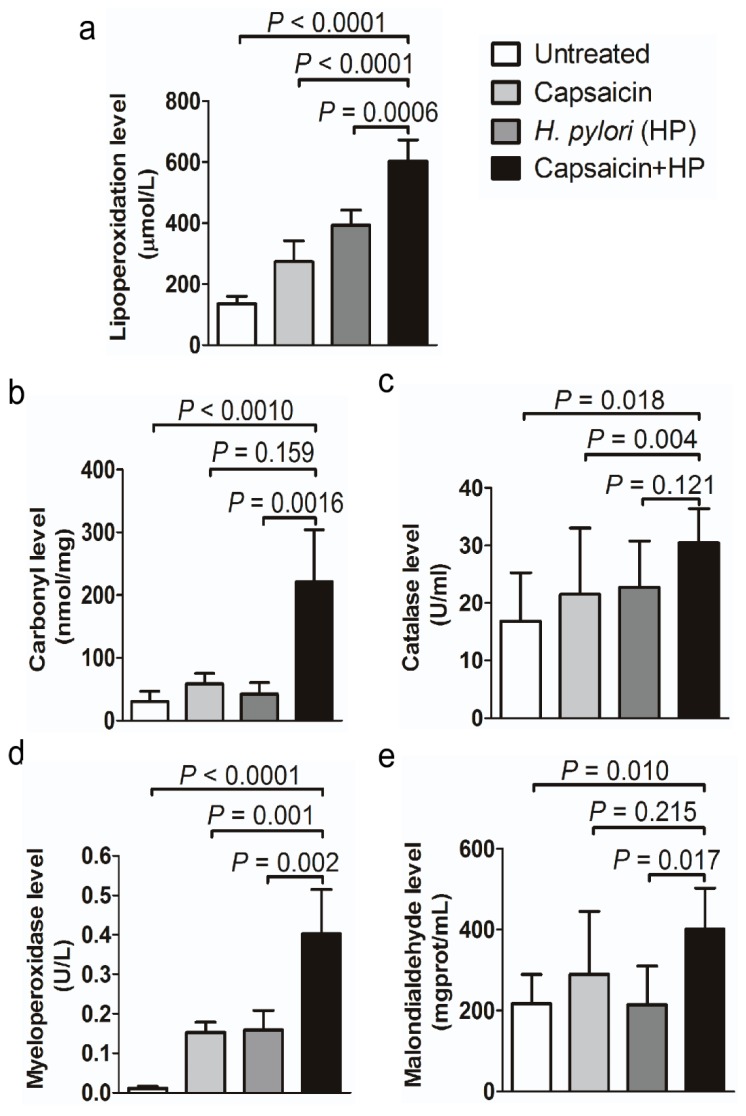
The level of tissue damage in gastric mucosa from gastric disease mouse models. Biochemical activity of (**a**) lipoperoxidase (LPO), (**b**) carbonyl, (**c**) catalase, (**d**) myeloperoxidases (MPO), and (**e**) malondialdehyde (MDA) in mice. Mice exhibiting gastritis or tumorigenesis show higher biochemical activity of these parameters compared to mice treated with capsaicin or *H. pylori* only (*n* = 5/cohort).

The extent of gastric tissue damage was evaluated as well. We found that mice treated simultaneously with capsaicin and *H. pylori* exhibited tumor development. These mice displayed significantly higher activities of malondialdehyde (MDA; 401.100 ± 45.33 mg/mL; *p* < 0.01), myeloperoxidases (MPO; 0.402 ± 0.050 U/L; *p* < 0.0001), catalase (30.47 ± 2.645 U/mL; *p* = 0.018), carbonyl (220.4 ± 37.35 nmol/mg; *p* < 0.001), and lipoperoxidase (LPO; 601.6 ± 31.61 µmol/L; *p* < 0.0001) compared to mice treated with combination of *H. pylori* and capsaicin (Figure 3a–e). A combination of capsaicin and *H. pylori* infection increases peroxidation processes and leads to the production of superoxide radicals such as MDA, MPO, and hydrogen peroxide, which can form adducts with DNA and proteins. Subsequently, we proposed that *H. pylori* infection and capsaicin metabolites, including vanillylamine and vanillic acid and peroxides damage DNA, DNA-repair enzymes, and immune cell protein cytokines, resulting in gastric tissue damage [37,38,39]. 

### 2.5. The Mouse-Derived Xenograft Model (MDX) Confirmed the Efficacy of the H. pylori- and Capsaicin-Associated Gastric Cancer Model

**Figure 4:**
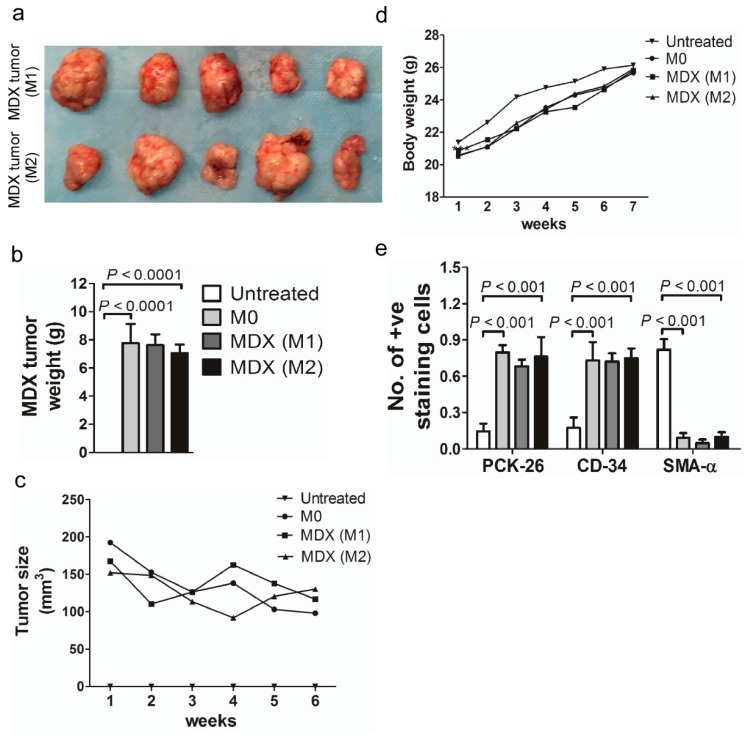
Mouse-derived xenograft model (MDX) to study the effect of combined treatment with capsaicin and *H. pylori*. (**a**) Macroscopic analyses of tumor tissue. Tumor images of first (M1) and second passage (M2) tumors showed a high growth rate in SCID mice. Tumors from mice treated with capsaicin and infected with *H. pylori* are represented as M0. The M1 and M2 tumor tissues were similar to M0 in (**b**) weight and (**c**) volume (mm^3^). (**d**) Bodyweight in grams measured per week. Mice were weighed before treatment (week 0) and then twice per week for 7 weeks. Mice with M0, M1, and M2 tumors lost weight compared to untreated control mice (*n* = 5/cohort). (**e**) Histological analyses of tumor tissues from mice treated with a combination of capsaicin and *H. pylori* (M0) with first (M1) and second passage (M2) xenografts. Histochemical analysis of PCK-26 and CD-34 in mouse tumor tissue (MDX) showed strong staining compared to the weak staining of SMA-α. A score of 1 denotes the highest staining and expression, whereas a score of 0 indicates negative staining or no expression. Quantitative immunohistochemistry of PCK-26 and CD-34 exhibited high scores in tumor tissues compared to a low score for SMA-α.

To further elucidate the effect of capsaicin intake and *H. pylori* infection on the development of gastric cancer, we used gastric tumors from mice treated with both *H. pylori* and capsaicin to develop a mouse xenograft model (MDX). We successfully developed the MDX model with tumors at the first (M1 = 100%) and second (M2 = 100%) passages (Figure 4a–d). Histological analysis further confirmed the tumorigenicity and gastric origin of this combination by showing high expression levels of cytokeratin (PCK-26; 0.797 ± 0.026, 0.682 ± 0.025, 0.761 ± 0.072), CD-34 (0.730 ± 0.067, 0.722 ± 0.030, 0.749 ± 0.035) and low expression levels of SMA-α (0.092 ± 0.017s, 0.046 ± 0.013, 0.099 ± 0.017) in M0, M1, and M2 tumor tissues, respectively (*p* < 0.001; Figure 4) [40,41]. PCK-26, CD-34, and SMA-α were used to confirm the tumorigenicity in the stomach. SMA-α and PCK-26 were investigated to detect the smooth muscle and non-epithelial (sarcomas) characteristics of the stomach, respectively, which showed the development of tumorigenicity. 

### 2.6. Capsaicin Dose in Mice Resembles Human Capsaicin Consumption and Mimics Stage-Dependent Gastric Cancer Development 

To apply our observations to humans, we optimized the mice’s intake to reflect human intake to determine the dose-dependent (0.01%–0.5% or 0.05–2.6 g/kg/day) effect of capsaicin. Mice treated with both *H. pylori* and capsaicin developed gastritis, leading eventually to cancer. To confirm this relationship in mice and to establish its relevance in humans, we conducted toxicity experiments in mice and optimized the capsaicin amount to determine its dose-dependent (0.01%–0.5% or 0.05–2.6 g/kg/day) effect. Mice treated with a combination of *H. pylori* infection and capsaicin (at a dose less than 0.06% or 0.312 g/kg/day but greater than 0.05% or 0.26 g/kg/day), which is relevant to a human dose of capsaicin (0.021–0.025 g/kg/day) induced a non-toxic effect. Notably, a dose greater than 0.05% or 0.26 g/kg/day induced significant toxicity and severe gastric inflammation. In contrast, a dose less than 0.05% or 0.26 g/kg/day did not cause any toxic effect or any significant gastric inflammation in mice (Tables S1 and S2). As a result, we selected a non-toxic, dose of 0.05% or 0.26 g/kg/day [8]. These results suggest that capsaicin dosage contributes considerably to the development of gastric inflammation and carcinogenesis. 

We also examined capsaicin intake, metabolism, and excretion. We found high blood assimilation of capsaicin in mice treated with capsaicin and *H. pylori* (0.023 ± 0.001 g/kg/day) compared to mice treated only with capsaicin (0.021 ± 0.001 g/kg/day). This observation corresponded with the low dissimilation of capsaicin of urine in the gastric tumor group (0.0003 ± 0.00001 g/kg/day) compared to the group treated with only capsaicin (0.0006 ± 0.0001 g/kg/day; Figure S3a). Similar results were found in human samples; gastric cancer patients (0.003 ± 0.0002 g/kg/day) and chronic gastritis patients (0.001 ± 0.0002 g/kg/day) showed high levels of capsaicin blood assimilation compared to healthy subjects (0.0005 ± 0.0001 g/kg/day; *p* < 0.0001; Figure S3b). These results were consistent with the role of capsaicin consumption in the development of physiological abnormalities and gastric cancer. High levels of capsaicin metabolites in urine following capsaicin consumption may be a sign of gastric tumor development as well. The risk for capsaicin-associated cancer is influenced by the expression of capsaicin metabolizing enzymes, such as 16-hydroxycapsaicin, 17-hydroxycapsaicin, and 16-, 17-dehydrocapsaicin, vanillylamine, and vanillin, which increase the risk for cancers [42,43,44]. As a result, we propose that capsaicin is a potential carcinogen. Ultimately, we developed a mouse model that utilizes a capsaicin dosage relevant to human capsaicin intake. With this model, gastric tumorigenesis is induced without toxicity in peripheral organs. As a result, this mouse model was used to study the effect of capsaicin and its metabolites on the development of gastric cancer. 

### 2.7. Capsaicin-Treated and H. pylori-Infected Mouse Tumors Showed Increased Pro-Inflammatory Cell Infiltration

**Figure 5:**
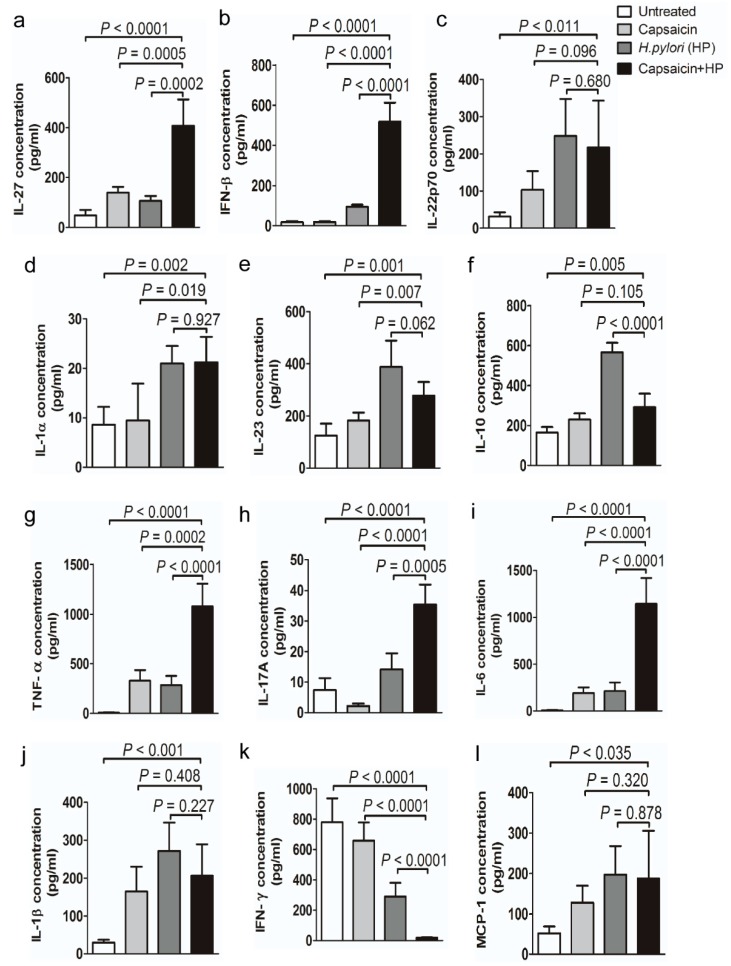
Analysis of cytokines in serum from gastric disease mouse models. Secretory profiles of pro-inflammatory cytokines (**a**) IL-27, (**b**) IFN-β, (**c**) IL-22P70, (**d**) IL-1α, (**e**) IL-23, (**f**) IL-10, (**g**) TNF-α, (**h**) IL-17A, (**i**) IL-6, (**j**) IL-1β, (**k**) IFN-γ, and (**l**) MCP-1 in mouse serum samples measured by multiplex magnetic bead array. Graphs show secreted protein expression relative to serum samples from untreated mice (*n* = 5/cohort).

Gastric inflammation leads to gastric tumor development. In our mouse model, the number of pro-inflammatory cell types was significantly increased (i.e., leukocyte infiltration), as observed by the numbers of activated macrophages (F4/80 positive) and neutrophils (myeloperoxidases, MPO) in mice exhibiting gastric tumors (0.746 ± 0.041 and 0.721 ± 0.064, respectively), compared to untreated mice or mice treated with only capsaicin or *H. pylori* (*p* < 0.0001; Figure S4a–b). Noting increased immunocyte infiltration, we analyzed several inflammatory-related chemokines and cytokines at the mRNA and protein (secretion) levels by using a customized multiplex magnetic bead array. In mice treated with capsaicin and *H. pylori*, gastric tumorigenesis was associated with augmented Th1 (IFN-γ, IL-1β, and GM-CSF) and Th2 (IL-10, IL-6, IL-13) cytokine expression (*p* < 0.001; Figure 5a–l). In particular, the pro-inflammatory cytokines IL-6 and IFN-γ showed high and low expression in mice with gastric cancer induced by treatment with capsaicin and *H. pylori*, respectively. Overall, *H. pylori* infection and capsaicin consumption produced great association towards IL-6 and IFN-γ expression. IL-6 and IFN-γ cytokines may play a critical role in the development of gastric inflammation and later gastric cancer.

### 2.8. DFMO Prevented H. pylori- and Capsaicin-Induced Gastric Cancers

**Figure 6:**
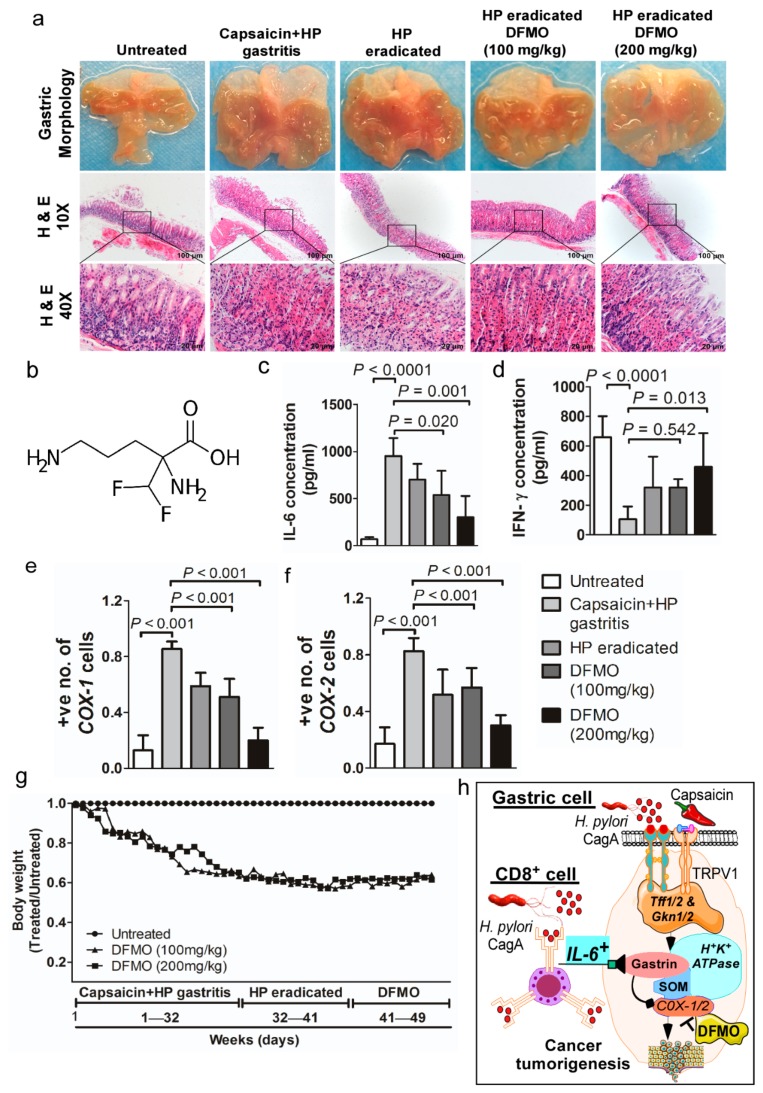
2-difluoromethylornithine (DFMO) preventive gastritis mouse model. (**a**) The gastric tissues from mice exhibiting gastritis induced by capsaicin and *H. pylori* or mice with *H. pylori* that was eradicated and treated with DFMO (100 or 200 mg/kg) did not show tumor development (*n* = 5/cohort). Histological sections were stained with H&E. The scale bars represent 100 μm (×10) and the scale bar in the inset images in each panel represents 20 μm (×40). (**b**) The chemical structure of DFMO. (**c**) IL-6, (**d**) IFN-γ secretory protein levels in mouse serum samples were measured by a multiplex magnetic bead array (*n* = 5). Quantitative immunohistochemistry of (**e**) COX-1 and (**f**) COX-2 expression in gastric tumors and gastritis tissues. (**g**) Effect of DFMO 100 and 200 mg/kg on mouse body weights. Body weights are shown as treated/untreated. Data are expressed as mean values ± SEM (*n* = 5/cohort). (**h**) A schematic drawing showing the proposed mechanism of capsaicin consumption combined with *H. pylori* infection in the development and progression of gastritis to gastric tumorigenesis.

We examined the role of IL-6 and IFN-γ in gastric cancer prevention by using the anti-inflammatory agent DFMO (scheme illustrated in Figure 6h). We administered capsaicin and *H. pylori* to mice, followed by DFMO treatment. Macroscopic morphometric analysis revealed that DFMO (100 and 200 mg/kg) inhibited gastric inflammation in a dose-dependent manner and prevented gastric tumorigenesis (Figure 6a,b). Histological analysis also showed multifocal elongation of the gastric pits, glandular atrophy, and a significant reduction in the glandular zone in atrophic foci. These changes were significant in mice treated with capsaicin and infected with *H. pylori*, exhibiting gastric inflammation (2.800 ± 0.200) compared to mice treated with DFMO (200 mg/kg; 1.400 ± 0.244) or untreated (0.6 ± 0.245; *p* = 0.002; Figure S5a–d). The anti-inflammatory potential of DFMO against gastritis and, therefore, against gastric cancer was evaluated by analyzing IL-6 and IFN-γ status. We observed a significant dose-dependent decrease and increase in the levels of pro-inflammatory cytokine IL-6 and IFN-γ for DFMO-treated mice compared to untreated mice exhibiting gastritis and to *H. pylori*-eradicated mice (*p* < 0.0001; Figure 6c,d), respectively. Moreover, other inflammatory cytokines and chemokines (IL-27, IFN-β, IL-1α, IL-23, IL-22P70, TNF-α, IL-17A, IL-10, IL-1β, and MCP-1) were not showed dose-dependent changed in DFMO-treated mice compared to gastritis mice group (Figure S6a–j). In contrast, COX-1 and COX-2 expression was also substantially reduced (*p* < 0.001; Figure 6e,f). DFMO-treated mice (100 or 200 mg/kg) showed significantly higher body weight compared to mice treated with a combination of capsaicin and *H. pylori* infection (Figure 6g). This result suggests that DFMO may prevent gastric tumorigenesis induced by a combination of capsaicin consumption and *H. pylori* infection. DFMO appears to act as an anti-inflammatory agent to prevent capsaicin-associated gastric cancer development by targeting COX-1 and COX-2 and by regulating IL-6 and IFN-γ expression. 

## 3. Discussion

Previous epidemiological and meta-analysis studies demonstrated a dose-dependent relationship between capsaicin consumption (30–80 g/day) and an increased risk of developing certain cancers, including stomach cancer [45,46,47]. Our study provides a biological rationale explaining high gastric cancer incidence in regions with high capsaicin consumption and *H. pylori* infection. Our mouse model confirms that capsaicin contributes to the induction of gastric inflammation leading to gastric cancer in the presence of *H. pylori* infection (Figure 1). 

Normal gastric mucosal development requires gastrin, somatostatin, and H+K+ATPase; however, all three are greatly diminished in mice with gastritis and tumorigenesis (Figure 2). These ablations affect gastric mucosal health and parietal cell activation. Moreover, treatment with a combination of capsaicin and *H. pylori* infection leads to increased peroxidation. This observation suggests that stimulation of gastric inflammation and gastric tumorigenesis involves the production of inflammation-inducing proteins and cytokines (Figure 3). Thus, capsaicin and *H. pylori* synergistically contribute to accelerated loss of differentiated epithelial cell types, leading to chronic atrophic gastritis and gastric tumorigenesis. 

We propose that *H. pylori* regulate pro-inflammatory cytokine production and release of IL-6, IFN-γ, IL-17A, IL-1β, IL-27, and TNF-α. This cytokine release causes gastric tissue damage leading to activation of inflammatory cancer signaling pathways (Figure 5). The outcome of IL-6 stimulation suggests that pre-existing gastric immunopathology accelerates the loss of differentiated epithelial cell types, leading to profound glandular atrophy and gastric tumorigenesis. In fact, IL-6 stimulation with IFN-γ inhibition may promote the shift from gastric inflammation to gastric carcinogenesis in mice administered both capsaicin and *H. pylori* (Figure 5). As a result, IL-6 inhibitors can serve as a therapy against gastric cancer, in addition to lowered capsaicin intake and pathogen clearance. Gastritis patients with high IL-6 levels may also benefit from IL-6 inhibition as a preventive measure against gastric carcinogenesis (Figure 5).

Tumorigenesis in *C57Balb/c* mice is attributed to IL-6 and IFN-γ deregulation in capsaicin-treated and *H. pylori*-infected mice. This deregulation leads to reduced expression of *Tff1* and *Tff2*, along with a reduced expression of putative tumor suppressor genes, *Gkn1* and *Gkn2*. *Tff1* and *Tff2* are important endogenous regulators of gastric homeostasis, and they showed loss of expression during capsaicin exposure and *H pylori* infection [25,27]. Accordingly, capsaicin and *H. pylori* are critical regulators of *Tff2* and upstream gastrokine gene expression. *Tff2* inhibition likely promotes tumor growth due to the loss of anti-proliferative gastrokine and *Tff1* genes. Additionally, the human GKN2 protein predominantly exists as a heterodimer with *Tff1* [28]; attenuated expression of either protein may potentiate their combined loss of function. Downregulation of both *Tff1* and *Tff2* expression also frequently occurs in gastric cancers displaying tumor suppressor function. However, loss of *Tff1* and *Tff2* promotes increased gastric inflammation and accelerates fundic atrophy with significant loss of parietal and chief cell lineages. This finding provides a mechanism for capsaicin-associated gastric cancer cell proliferation [29]. 

Our data suggest that combined capsaicin exposure and *H. pylori* infection modulate and imbalance Th1 and Th2 cytokine levels, leading to mucosal inflammation, inhibition of gastric acid secretion, and induction of fundic atrophy. Tumorigenesis in capsaicin- and *H. pylori*-treated mice was associated with augmented Th1 cytokine release, particularly IL-6, along with attenuated Th2 cytokine expression. Th1 cytokines like IL-6 promote chronic atrophic gastritis and predisposition to tumorigenesis through inhibition of gastrin and somatostatin, as indicated earlier (Figure 5). In combination, capsaicin and *H. pylori* provide a gastric cancer development mechanism in which local immune responses modulate and decrease essential gastric hormones. 

Here, the selected anti-inflammatory agent, DFMO (2-difluoromethylornithine) inhibits ornithine decarboxylase (ODC) activity, an enzyme catalyzing a rate-limiting step of polyamine biosynthesis. ODC is necessary for entry into and progression through the cell cycle; additionally, Selvakumaran et al. demonstrated that ODC is a transcriptional target of the *c-myc* oncogene [48]. In our mouse model, DFMO acts by inhibiting IL-6 and promoting IFN-γ cytokine release (Figure 6), plus it enhances antitumor CD8^+^ T-cell infiltration and augments adoptive T-cell therapy. Furthermore, DFMO impairs the suppressive activity of myeloid-derived suppressor cells (MDSC), reverses tumor-induced immunosuppressive mechanisms, and invokes a measurable antitumor immune response [22,49]. Consequently, DFMO treatment may serve as a preventive measure against gastric tumorigenesis induced by capsaicin consumption and *H. pylori* infection.

## 4. Methods

### 4.1. Preparation of H. pylori Strains

*Helicobacter pylori* (*H. pylori*, Sydney strain or SS1) was provided by Professor Young-Joon Surh (Seoul National University, Seoul, South Korea). *H. pylori* SS1 was grown on Columbia Agar (Oxoid, Basingstoke, Hampshire, England, Cat number CM0331) plates for 48 h at 37 °C under microaerophilic conditions on 7% lysed horse blood (Solarbio, Beijing Solarbio Science & Technology, Beijing, China Cat number S9050) agar and antibiotics, including amphotericin B (1.5 μg/mL; Solarbio, Cat number: A8250), trimethoprim (1.25 μg/mL) (Tichea Chemical Industry Development Co, Shanghai, China, Product code T2286), and vancomycin (2.5 μg/mL) (Solarbio, Cat number: V8050). Identification was performed by Gram staining (Solarbio, Cat number G1060) and testing for urease (Nanjing Jiancheng Bioengineering Institute, Nanjing, China; Cat number C013-2), catalase (Nanjing Jiancheng Bioengineering Institute, Cat number A007-1), and molecular markers.

### 4.2. Animal Model Experimental Design

Mice were eight-week-old male C57-BL/6J-219 mice (18–20 g; Beijing Vital River Laboratory, Beijing, China) that were free of *Helicobacter* spp., *Citrobacter rodentium*, and *Salmonella* spp. Mice were maintained under specific pathogen-free conditions and fed sterilized commercial pellet diets (Beijing HFK Bioscience, Beijing, China) and sterile water ad libitum and housed in an air-conditioned biohazard room at a controlled temperature of 24 °C, under a 12-h light/dark cycle. The experiments were performed after protocol approval by the ethics committee of China-US (Henan) Hormel Cancer Institute, Henan, China, and were conducted in accordance with the current guidelines for laboratory animal care. C57-BL/6J-219 (Access No: CUHCI2015011) were randomly assigned to 5 groups of 10 animals each. After one week of acclimation, mice were administered capsaicin (0.05% or 0.2 g/kg/day) in dietary food for two weeks prior to infection with *H. pylori*, and capsaicin administration was continued throughout the experiment. To facilitate *H. pylori* colonization, pantoprazole (20 mg/kg) was administered by gavage 3 times to lower gastric acidity. Each mouse was administered a suspension of the *H. pylori* SS1 strain containing 10^8^ CFUs/mL by gavage 3 times per week. Mice (*n* = 5) were euthanized on consecutive weeks of 4, 8, 12, 16, 20, 24, 28, 32, 36, 40, 44, 48, and 52 weeks. A control group was maintained without any treatment and was euthanized at the same time as the experimental group. In another mouse model, doses of capsaicin ranging from 0.5–5.0 g/kg/day were administered to mice (*n* = 5) to determine the optimal capsaicin dose to induce gastric tumorigenesis. In addition, after confirmation of gastritis development at 32 weeks after *H. pylori* infection, a DFMO-treated gastritis mouse model was developed (Access No: CUHCI2016015). *H. pylori* were eradicated by administering by gavage a triple regimen of omeprazole (400 μmoL/kg/day; Jiangsu Pengayao Pharmaceutical. Co., Yinxing, Jiangsu, China), metronidazole oral suspension (14.2 mg/kg/day; Guizhou Feiyuling Pharmaceutical. Co., Guizhou, China), and clarithromycin granules in oral suspension (7.15 mg/kg/day; Xiuzheng Pharmaceutical Co. Changchun, Jilin, China) twice daily for 7 days with a 30–60 min interval between omeprazole and antibiotics. Persistence of gastric inflammation after successful eradication of *H. pylori* was analyzed after two months (41 weeks). C57-BL/6J-219 mice that still had *H. pylori* after the eradication therapy were excluded from the analysis. *H. pylori* colonization and eradication were confirmed by calculating the Cfu/gram of stomach and colonization assay (urease test) (Figure S7a,b). *H. pylori*-eradicated mice were divided into two groups based on DFMO dose (100 and 200 mg/kg groups; 49 weeks; Sigma Aldrich, St. Louis, MO, USA, Cat number 17378). Mice were administered a suspension of DFMO by gavage 3 times a week for 2 months. A toxicity assay was performed to optimize the amount of consumption. Mice (*n* = 10) were administered different amounts of capsaicin (0.01%–0.5% or 0.05–2.6 g/kg/day) up to 3 weeks, and mouse survival rate was analyzed. 

Ethics approval and consent to participate: The institutional review board at the China–US (Henan) Cancer Institute approved the study (Access No: CUHCI2015011, CUHCI2016015 and CUHCI2016024). Subjects provided informed consent to be included.

### 4.3. Gastric Sample Preparation

Mice were euthanized, and their stomachs excised and gently rinsed with cold saline. For gastric tumor phenotype analyses, stomachs were opened along the greater curvature and spread out on a polypropylene sheet (Jiangsu Jiangyin Jinfeng Textile Co., Jiangsu, China). The area (mm^2^) of the mucosal erosive lesions was measured, and tumors were macroscopically identified in the gastric mucosae by micro-dissection of gastric tumor tissue. The anterior wall of the pyloric antrum contains the pyloric glands and gastric epithelial cells were cut into several linear strips for quantitative enzyme activity, DNA, RNA, protein, and histological analyses. Other peripheral organs were also collected, including brain, lung, liver, kidney, spleen, colon, and heart, in order to analyze the toxic effect of capsaicin consumption by H&E-staining. 

### 4.4. Patients and Gastroendoscopy

All gastric patients were subjected to gastroendoscopy and examination in the Second Affiliated Zhengzhou University Hospital and Henan Cancer Hospital (Zhengzhou, Henan, China). Blood, urine samples, and tissue biopsies were obtained from consenting patients from the antral and corpus portions of the stomach during gastrointestinal endoscopy and gastric surgery. The patients who donated the primary tumors were completely informed and provided written consent (Access No: CUHCI2015009). 

### 4.5. Morphometric and Histological Analysis 

Morphometric analysis was performed using Image-J software ((http://rsb.info.nih.gov/ij/index.html); National Institutes of Health, Bethesda, MD, USA). For the histological analysis, tissues examined consisted of a section of the stomach taken from the greater curvature and cut from the fundus to the proximal end of the corpus. Longitudinally bisected half stomachs were retained in buffered formaldehyde sections of 6 mm and stained with H&E. These samples were scored depending on the severity of (i) superficial regional disruption of the gastric gland with epithelial cell loss, (ii) neutrophil infiltration & mononuclear cell infiltration (inflammatory cells), (iii) glandular zone reduction, and (iv) functional atrophy (loss of parietal and chief cells), using a scale ranging from 0 to 4 (0: none; 1: weak; 2: mild; 3: moderate; and 4: severe) for each criterion. The sections were assessed by an experienced pathologist without knowledge of the treatments. The presence of mononuclear cell infiltration and polymorphonuclear (PMN) cell infiltration indicated chronic gastritis. The length of the glandular zone, primarily composed of parietal cells, was measured as a proportion of total mucosal thickness. Antral tumor tissues were analyzed with measurement of tumor volume and compared with the controls. 

### 4.6. Immunohistochemistry (IHC) 

Paraffin-embedded gastric tissues were analyzed by immunohistochemistry (IHC). Serial sections (4–6 mm each) were deparaffinized in xylene and rehydrated in a capsaicin concentration gradient and evaluated with antibodies to detect *H. pylori* (1:100), pepsinogen I and II (1:100 each), MPO (1:100), F4/80 (1:100), CD-34 (1:100), PCK-26 (1:100), and SMA-α (1:100). Sections were subsequently incubated with their respective secondary antibodies for 30 min at room temperature. The signal was visualized with peroxidase-labeled streptavidin complexes by DAB, and the sections were briefly counterstained with hematoxylin. The immunohistochemical localization pattern was also recorded by digital imaging (Nikon Ti-DS, Tokyo Japan). The ImageScope (11.1.1.752) software program was used, and the labeling index was calculated as a percentage of positive cells relative to the total number of counted cells.

### 4.7. Quantitative Enzyme and Activity Assays 

Quantitative enzyme assays included measurement of malondialdehyde (MDA; Cat number A003-1), myeloperoxidase (MPO; Cat number A044), carbonyl (Cat number A087), lipoperoxidase (LPO), catalase (Cat number A007-1), and urease (Cat number C013-2) and were performed according to the manufacturer’s instructions (Nanjing Jiancheng Bioengineering Institute, Nanjing, China).

### 4.8. Real-Time RT-PCR

Total RNA was extracted using a commercial RNA extraction kit (Ambion by Life Technologies, Carlsbad, CA, USA), and cDNA was synthesized by amfiRivert cDNA synthesis platinum master mix (GenDEPOT, Katy, TX, USA, Cat number R5600-200). Real-time PCR (qRT–PCR) was conducted using a 7500 FastDX (Applied Biosystems, MA, USA) and the Power SYBR green PCR master mix (Applied biosystem, Warrington WA1 4SR, UK, Cat number 4367659). Primer IDs and sequences are shown in Table S3. 

### 4.9. Cytokine and Chemokine Protein Measurement Using a Multiplex Bead Array

Cytokine and chemokine protein levels in mouse (Cat number 740446) and human (Cat number 740118) serum were measured using a multiplex magnetic bead array kit (customized by BioLegend LEGENDplex, San Diego, CA, USA). The multiplex bead arrays were performed according to the manufacturer’s instructions with a minimum detectable concentration varying from 0.96–11.27 pg/mL. The Legendplex (version: 7.0) software program was used to analyze the FACS data, and the cytokine concentrations were calculated in pg/mL against standard values.

### 4.10. H. pylori CFU Quantification within Mouse Stomachs 

After 1, 3, or 6 weeks, mice were euthanized. Stomachs were halved longitudinally along the greater and lesser curvatures and rinsed in sterile phosphate-buffered saline. Each half was manually disrupted on ice in 750 μL of Iso-Sensitest (Oxoid, Basingstoke, UK) broth/15% glycerol. Cells were serially diluted and plated on Columbia blood agar base plates (Oxoid, Basingstoke, UK, Cat number CM0331), supplemented with 10% defibrinated horse blood (Solarbio, Beijing, CRP, Cat number S9050), and Dent supplement (Oxoid, Basingstoke, UK, Cat number SR0147E).

### 4.11. Urine Collection and Measurement (Metabolic Cage Experiments)

Capsaicin metabolism and urinary flow rate were determined by placing mice individually in metabolic cages. Mice were allowed a 3-day habituation period to adapt to the environment. Later, food and water intake, urinary flow rate, and body weight were recorded every day. Subsequently, a 12 h collection (9 p.m. to 9 a.m.) of urine was performed to obtain the urinary parameters, including volume, pH, and color. Data were analyzed for capsaicin intake, metabolism, and excretion. Capsaicin content in the serum and urine samples from mice and humans was measured according to the Capsaicin ELISA Commercial kit’s instructions (Shanghai Jianglai Biotechnology, Shanghai, P.R.C, Cat number JL22882).

### 4.12. Mouse-Derived Xenograft Model

A mouse-derived xenograft model was developed from gastric tumor tissue of mice treated with capsaicin (Access No: CUHCI2016024). Tissue samples were placed in a Petri plate containing phosphate-buffered saline (PBS) with 0.1 mL of penicillin (1 × 10^5^ U/mL; North China Pharmaceutical, Hebei, China), gentamycin (8 × 10^3^ U/mL; Kaifeng Pharmaceutical Co., Henan, China), and streptomycin (1 × 10^5^ U/mL; North China Pharmaceutical, Hebei, China). The tissues were divided into three parts. One portion was implanted into CB17 SCID mice purchased from Beijing Vital River Laboratory (Beijing, China). The second portion was fixed in 10% formalin, and the third portion was used for protein extraction. The mice were anesthetized using 0.3 mL of 0.4% (*w/v*) pentobarbital sodium (150 μL/10 g; Sinopharm Chemical Reagent, Guangdong, China) for every 20 g of body weight. Then mice were subcutaneously implanted with tissues weighing 0.10–0.12 g and measuring ~3 mm. Animals were monitored periodically for their weight and tumor growth. A second passage was performed, and the same protocol was followed as described above. 

### 4.13. Statistical Analysis

The experiments were randomized and investigators were blinded to histological examination during all experiments. All statistical analyses were performed using Graphpad Prism 5.0 software (San Diego, CA, USA), with differences between groups considered significant with a *p*-value < 0.001. Data are presented as mean values ± SEM. Histopathological scores and all other experimental data were compared using a *t*-test (two-sided) or one-way analysis of variance (ANOVA) followed by (post hoc) Newman–Keuls multiple and Tukey multiple comparison tests. The discriminatory ability of markers for gastric cancer was evaluated by receiver operating characteristics Curve (ROC), providing the area under the curve (AUC). All tests were two-sided, and *p*-values ≤ 0.01 were considered statistically significant. Statistical software IBM SPSS 20.0 (SPSS Inc., Chicago, IL, USA) and R program package (Wirtschafts Universität, Wien, Austria) were used to perform these analyses.

## 5. Conclusions

This study describes mechanisms governing the induction and progression of chronic gastritis to gastric cancer under the inflammatory triggers of *H. pylori* infection and capsaicin exposure. This study also proposes DFMO treatment to prevent gastric cancer through pro-inflammatory IL-6 cytokine inhibition, resulting in diminished gastric inflammation. This study is the first to provide experimental animal evidence showing gastric cancer prevention by DFMO treatment in mice with capsaicin consumption and *H. pylori* infection. In the future, these mouse models can also facilitate further studies into the molecular mechanisms of chronic gastritis and gastric cancer.

## Supplementary Materials

All data needed to evaluate the conclusions of this paper are present in the paper and/or Supplementary Materials. The supplementry data underlying Figure S1a–d: Gastric histology scores in gastric disease mice models, Figure S2a–d: Gastric tumor suppressor gene expression generally decreases in mice treated with both capsaicin and *H pylori*, Figure S3a,b: Capsaicin consumption in the development of gastric tumorigenesis, Figure S4a–d: Analysis of inflammatory mediators in tissues from the gastric disease mouse models and Figure S5a–d: Gastric histology scores in DFMO preventive gastric disease mice models, Figure S6a–j: Analysis of cytokines in serum from DFMO treated cancer preventive mouse models, Figure S7: Quantification of *H. pylori* (CFU/g of stomach) and *H. pylori* urease expression level in gastric mucosa, Table S1: Toxicity assay to optimize the capsaicin concentration for the development of gastritis leading to gastric cancer mice model, Table S2: Dose dependent effect of capsaicin in the development of gastritis to cancer mice model, and Table S3: qRT–PCR primer sequences (mouse) are provided as supplementary data files.

## Abbreviations

Gkns	Gastrokines
*H. pylori*	*Helicobacter pylori*
IARC	International Agency of Research on Cancer
IFN	interferon
IL	interleukin
LPO	lipoperoxidase
MDA	malondialdehyde
MDX	mouse xenograft model
MPO	myeloperoxidases
SMA-α	smooth muscle antigen
*Tff1*	*Trefoil factor1*
*Tff2*	*Trefoil factor2*
TNF	tumor necrosis factor
TSGs	tumor suppressor genes
WHO	World Health Organization
