# Supplementary material for: Induction and Prevention of Gastric Cancer with Combined Helicobacter Pylori and Capsaicin Administration and DFMO Treatment, Respectively
Source: Cancers (Basel). 2020 Mar 28;12(4):816. doi: 10.3390/cancers12040816 (PMC7226438; doi:10.3390/cancers12040816)
Supplement: Supplementary file 1 [file cancers-12-00816-s001.zip › cancers-754322-suppl-v1-final/cancers-754322-suppl-v1.pdf]

# Supplementary Materials: Induction and prevention of gastric cancer with combined *Helicobacter pylori* and capsaicin administration and DFMO treatment, respectively

**Faisal Aziz, Mingxia Xin, Yunfeng Gao, Abhijit Chakroborty, Imran Khan, Josh Monts, Kjersten Monson, Ann M. Bode and Zigang Dong**

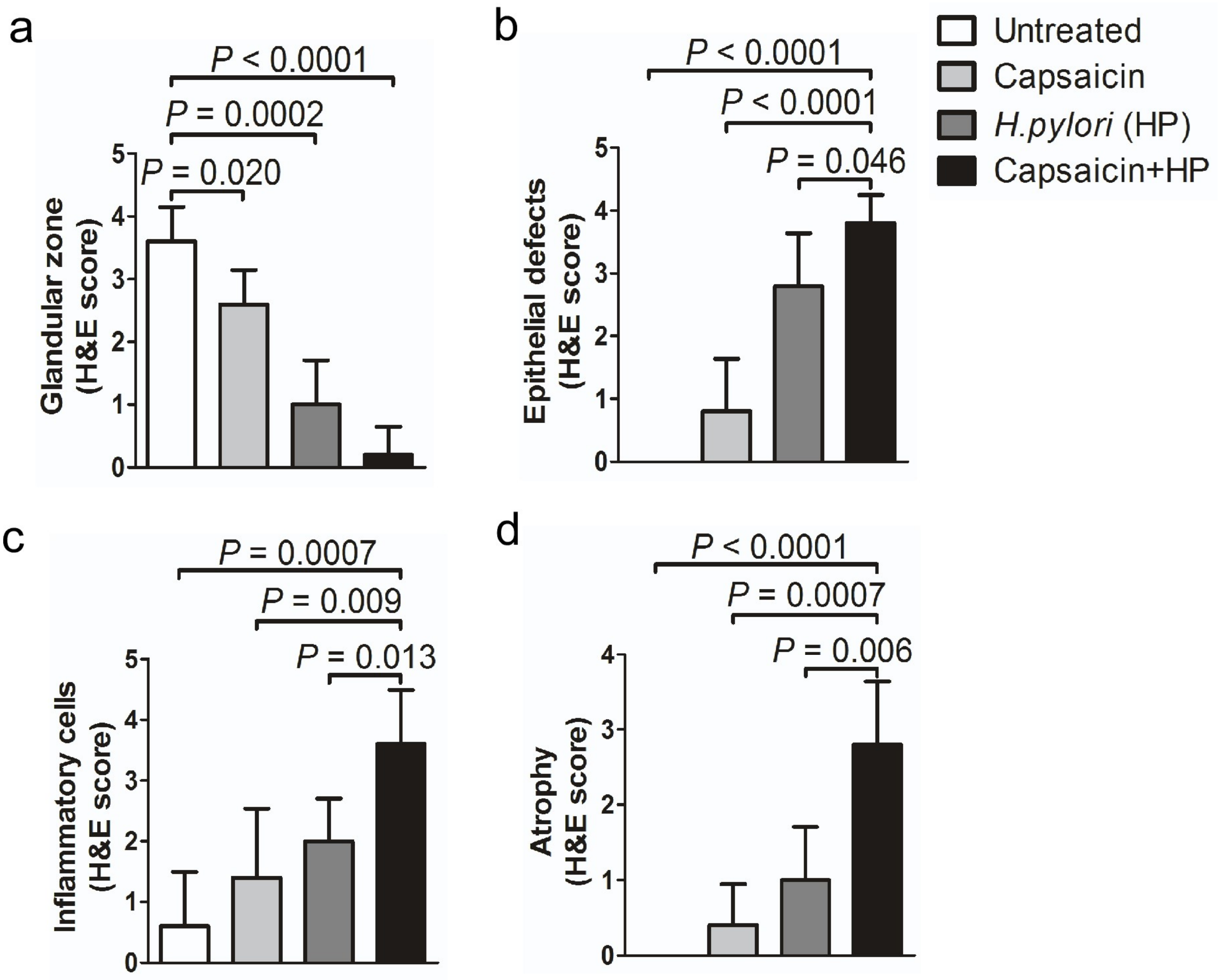


**Figure S1.** Gastric histology scores in gastric disease mice models. Individual lesion scores of every mouse were compared for (**a**) glandular zone, (**b**) epithelial defects, (**c**) atrophy, and (**d**) infiltration of inflammatory cells; ($n$ = 5/cohort). Mice exhibiting gastric tumorigenesis induced by combined *H. pylori* and capsaicin treatment showed a significantly higher score (greater severity) compared to mice exhibiting gastritis with no tumor. Mice treated with only capsaicin or only *H. pylori* showed milder pathology, and the untreated group had normal histology.

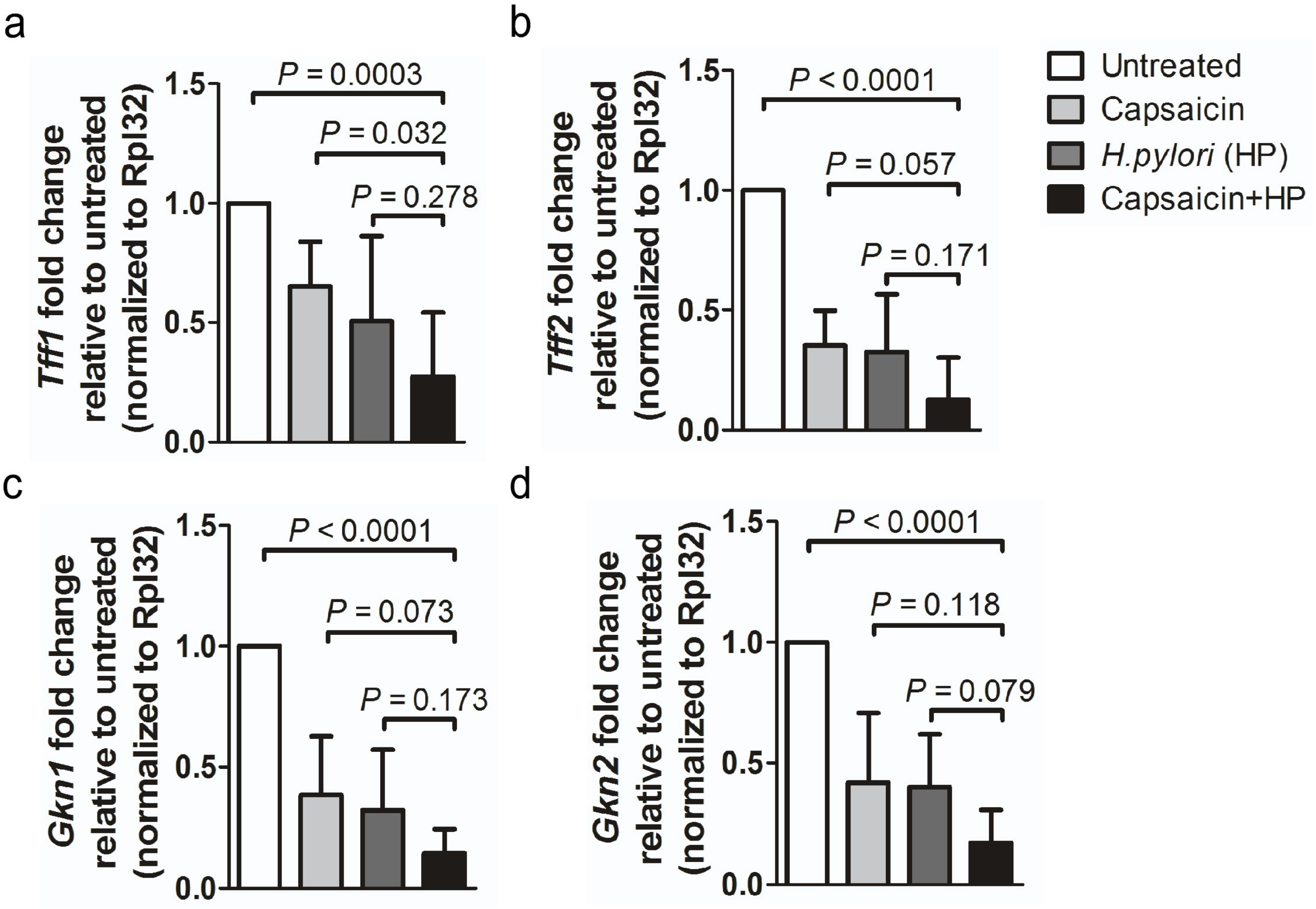


**Figure S2.** Gastric tumor suppressor gene expression generally decreases in mice treated with both capsaicin and *H pylori*. mRNA analysis of (**a**) *Tff1*, (**b**) *Tff2*, (**c**) *Gkn1*, and (**d**) *Gkn2* by qPCR. Histograms show the average fold change in mRNA expression compared to untreated mice ($n$ = 5/cohort).

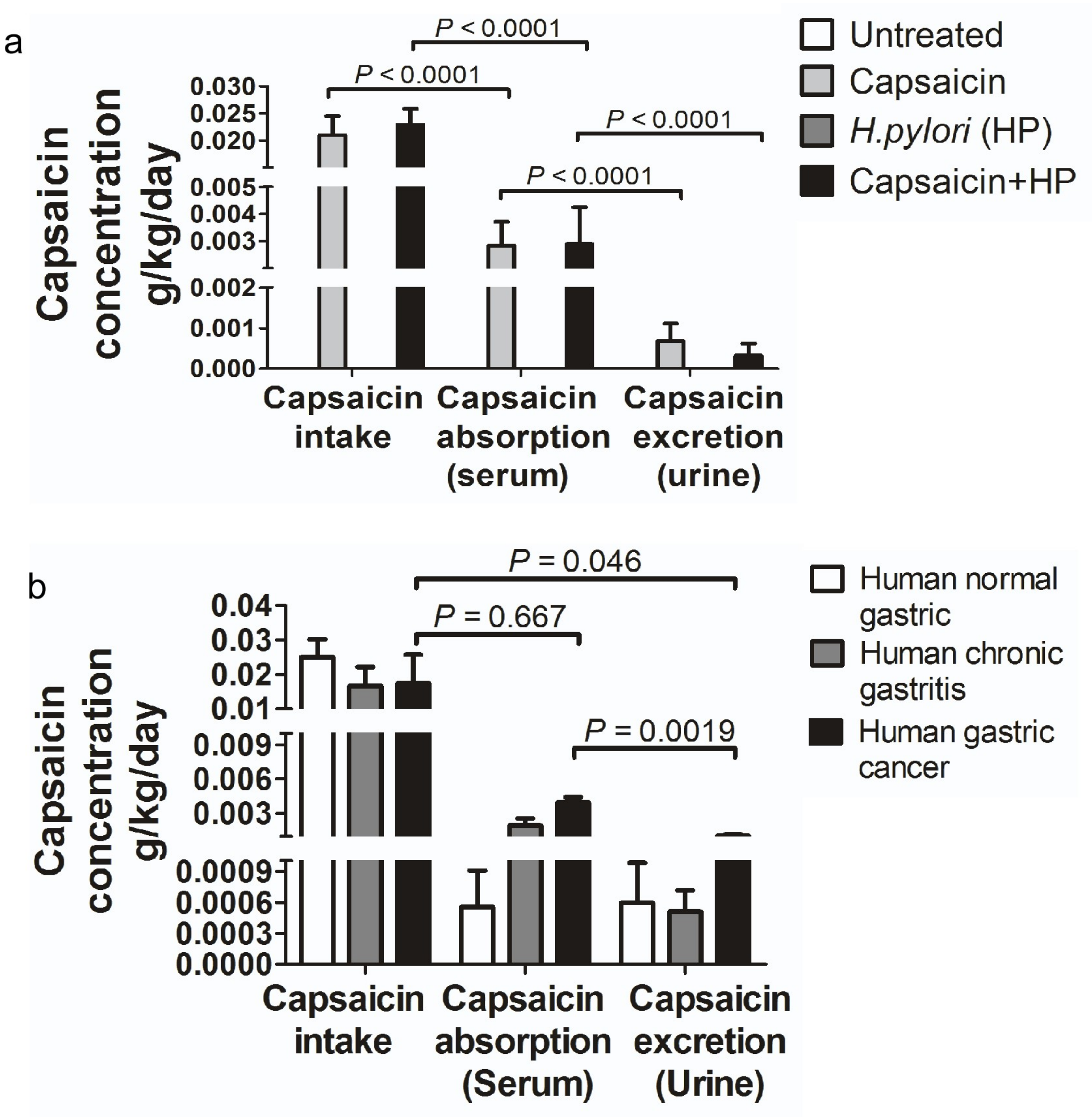


**Figure S3.** Capsaicin consumption in the development of gastric tumorigenesis. Association of capsaicin intake, metabolism, and excretion in mouse and human gastric disease samples. Capsaicin concentration in (**a**) mouse and (**b**) human ($n$ = 10) serum and urine samples was used to calculate capsaicin intake, absorption (serum), and excretion (urine). Analysis of urine samples from the gastric disease mouse models**.**

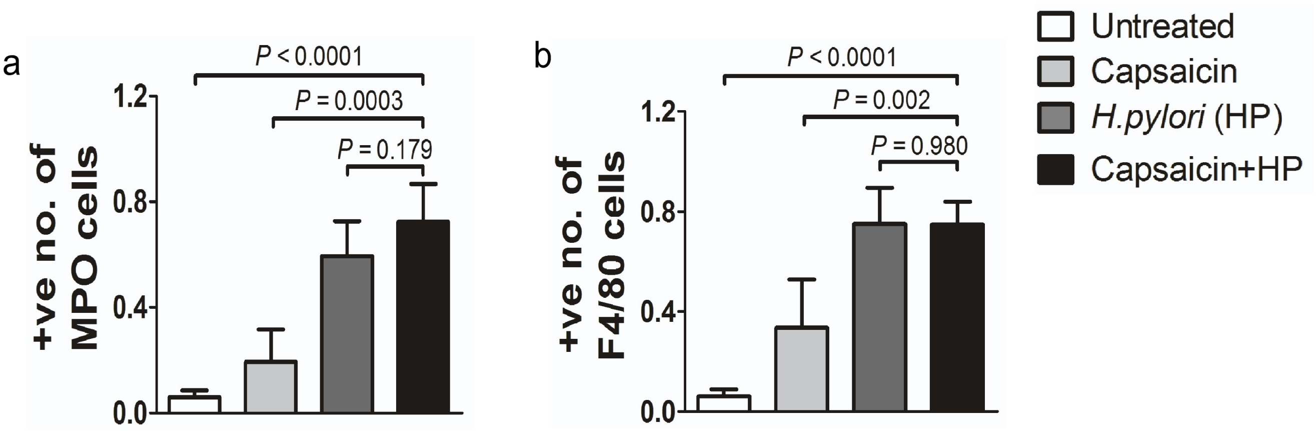


**Figure S4.** Analysis of inflammatory mediators in tissues from the gastric disease mouse models. Immunohistochemical analysis of MPO and F4/80 expression in gastric tissues of the various mouse models. Quantitative immunohistochemistry to detect (**a**) MPO and (**b**) F4/80 expression revealed a higher score in tumor tissue compared to tissues from mice treated with capsaicin or *H. pylori* only. Staining intensity was weaker in untreated gastric tissue compared to gastric tumor tissue. The scale bars represent 100 μm (10×), and the scale bar in the inset images represents 20 μm (40×). A score of 1 denotes the highest staining or expression levels, whereas a score of 0 indicates negative staining.

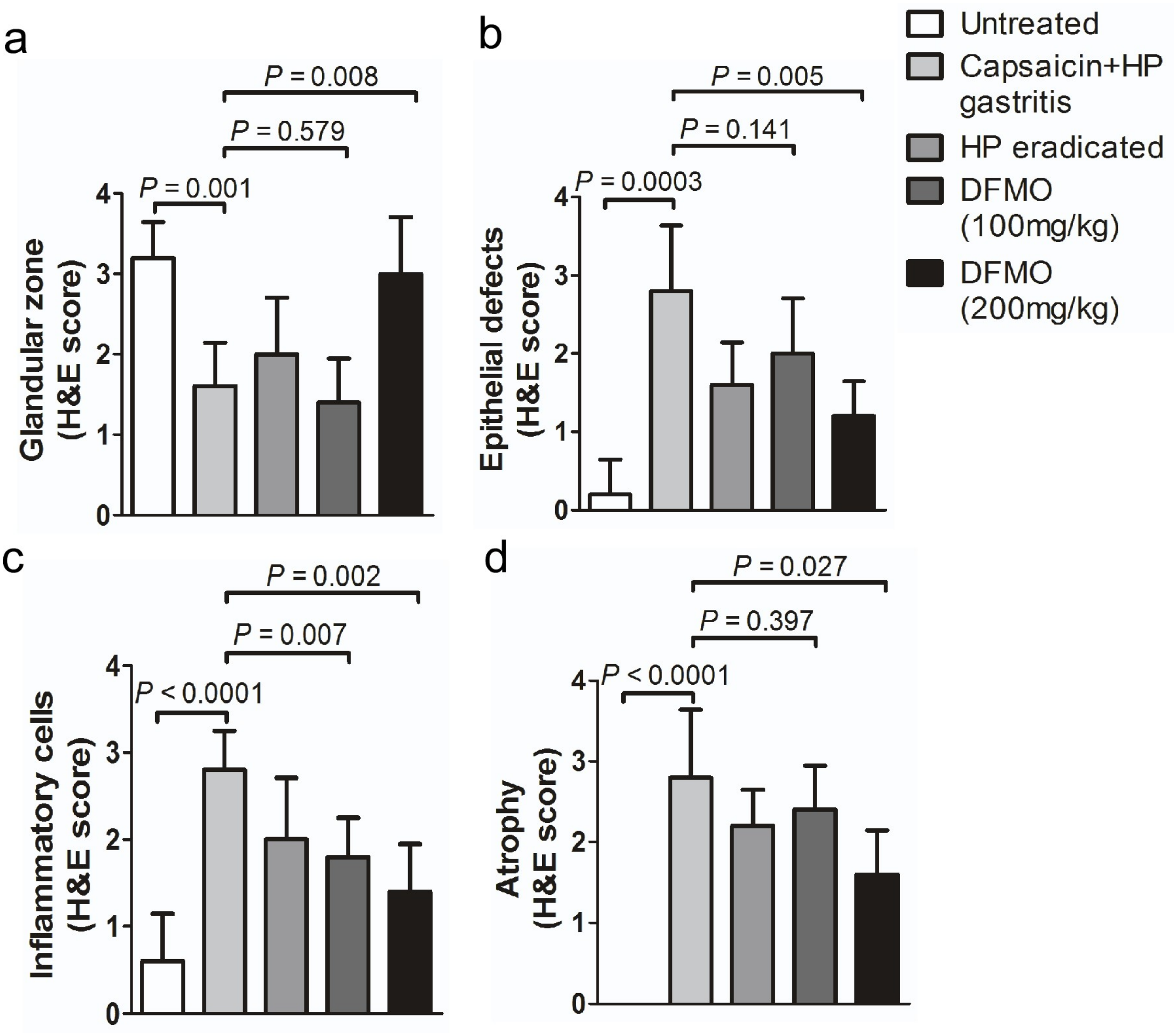


**Figure S5.** Gastric histology scores in DFMO preventive gastric disease mouse models. Individual lesion scores of every mouse were compared for (**a**) glandular zone, (**b**) epithelial defects, (**c**) infiltration of inflammatory cells, and (**d**) atrophy; ($n$ = 5/cohort). Mice exhibiting gastric tumorigenesis induced by combined *H. pylori* and capsaicin treatment showed a significantly higher score (greater severity) compared to mice treated with DFMO exhibiting low gastric inflammation gastritis with no tumor. Mice eradicated with *H. pylori* showed milder pathology, and the untreated group had normal histology.

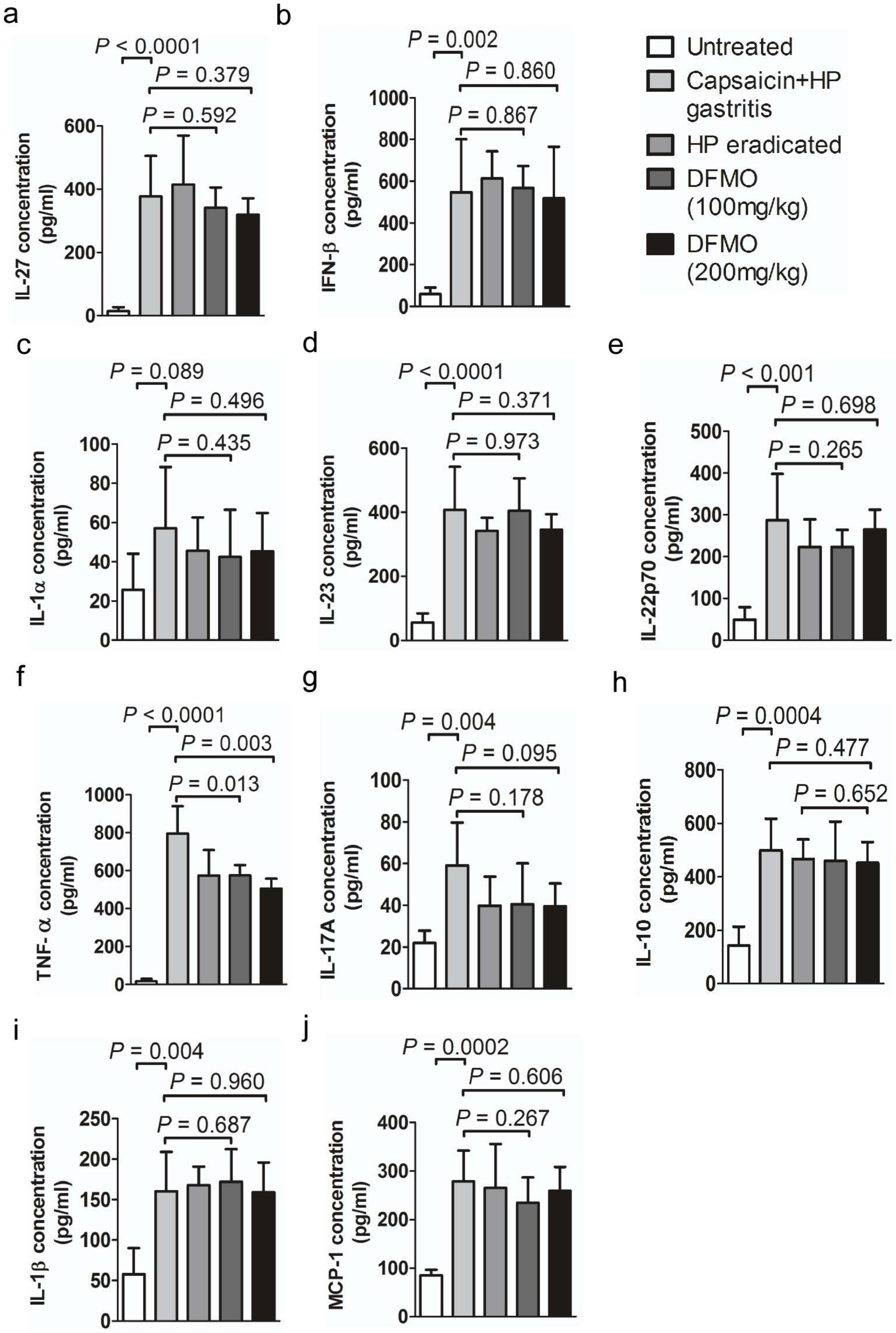
a
P < 0.0001
P = 0.379
P = 0.592
IL-27 concentration (pg/ml)
b
P = 0.002
P = 0.860
P = 0.867
IFN-β concentration (pg/ml)
Untreated
Capsaicin+HP gastritis
HP eradicated
DFMO (100mg/kg)
DFMO (200mg/kg)
c
P = 0.089
P = 0.496
P = 0.435
IL-1α concentration (pg/ml)
d
P < 0.0001
P = 0.371
P = 0.973
IL-23 concentration (pg/ml)
e
P < 0.001
P = 0.698
P = 0.265
IL-22p70 concentration (pg/ml)
f
P < 0.0001
P = 0.003
P = 0.013
TNF- α concentration (pg/ml)
g
P = 0.004
P = 0.095
P = 0.178
IL-17A concentration (pg/ml)
h
P = 0.0004
P = 0.477
P = 0.652
IL-10 concentration (pg/ml)
i
P = 0.004
P = 0.960
P = 0.687
IL-1β concentration (pg/ml)
j
P = 0.0002
P = 0.606
P = 0.267
MCP-1 concentration (pg/ml)

**Figure S6.** Analysis of cytokines in serum from DFMO treated cancer preventive mouse models. Secretory profiles of pro-inflammatory cytokines (**a**) IL-27, (**b**) IFN-β, (**c**) IL-1α, (**d**) IL-23, (**e**) IL-22P70, (**f**) TNF-α, (**g**) IL-17A, (**h**) IL-10, (**i**) IL-1β and (**j**) MCP-1 in mouse serum samples measured by multiplex magnetic bead array. Graphs show secreted protein expression relative to serum samples from untreated and capsaicin+*H. pylori* gastritis mice (*n* = 5/cohort).

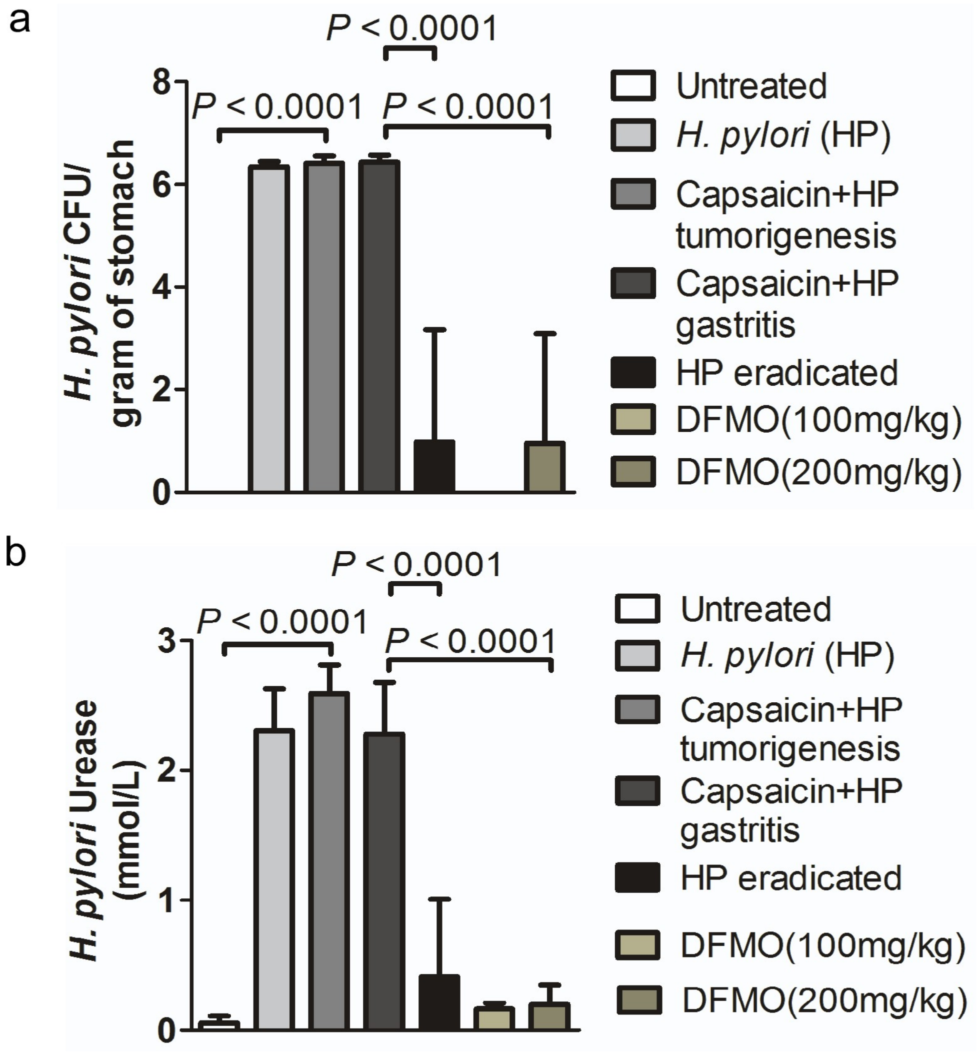


**Figure S7.** Quantification of (**a**) *H. pylori* (CFU/g of stomach) and (**b**) *H. pylori* urease expression level in gastric mucosa from mice treated with *H. pylori*, mice treated with both alcohol and *H. pylori (tumorigenesis and gastritis)* showed higher urease activity and CFU compared to mice eradicated with *H. pylori as well as treated with DFMO* (*n* = 5/cohort).

**Table S1.** Toxicity assay to optimize the capsaicin concentration for the development of gastritis leading to gastric cancer mice model.

| **Capsaicin concentration (%)** | **0.01** | **0.02** | **0.03** | **0.04** | **0.05** | **0.06** | **0.08** | **0.1** | **0.25** | **0.5** |
|---|---|---|---|---|---|---|---|---|---|---|
| **Capsaicin concentration (g.kg/day)** | 0.052 | 0.104 | 0.156 | 0.208 | 0.26 | 0.312 | 0.416 | 0.52 | 1.3 | 2.6 |

| | | | | | | | | | | |
|---|---|---|---|---|---|---|---|---|---|---|
| **Mice dead at 1st week 29-7-2016** | 0 | 0 | 0 | 0 | 0 | 0 | 0 | 3 | 5 | 6 |
| **Mice dead at 2nd week 5-8-2016** | 0 | 0 | 0 | 0 | 0 | 0 | 4 | 3 | 3 | 3 |
| **Mice dead at 3rd week 12-8-2016** | 0 | 0 | 0 | 0 | 0 | 6 | 5 | 2 | 1 | 10 |

**Table S2.** Dose dependent effect of capsaicin in the development of gastritis to cancer mouse model.

| **Alcohol concentration (g/kg)** | **0.01** | **0.02** | **0.03** | **0.04** | **0.05** | **0.06** | **0.08** | **0.1** | **0.25** | **0.5** |
|---|---|---|---|---|---|---|---|---|---|---|
| **Morphological effect** | 0 | 0 | | 0 | 0 | 0 | Dark streak | Dark streak | Dark streak | Dark Streak |
| **Inflammation level** | - | - | - | + | ++ | +++ | ++++ | ++++ | ++++ | +++++ |
| **Epithelial damage** | - | - | - | - | - | +++ | +++ | ++++ | ++++ | +++++ |
| **Atrophy** | - | - | - | - | - | + | + | ++ | ++ | +++ |

**Table S3.** qRT-PCR primer sequences (Mouse).

| **Gene** | **Forward primer Sequence** | **Reverse primer sequence** |
|---|---|---|
| ***Tff1*** | 5’-AGAGGTTGCTGTTTTGATG-3’ | 5’-AGTCTGAGGGGTTGAACTG-3 |
| ***Tff2*** | 5’-CCCCACAACAGAAAGAAC-3’ | 5’-GGGCACTTCAAAGATCAG-3’ |
| ***Gkn1*** | 5’-CTTCAGGACCTCGATACAATGG-3’ | 5’-TTGAGTACAAAGGCTGGTTTGG-3’ |
| ***Gkn2*** | 5’-AATGTAGACGGAAGTGGACAGC-3’ | 5’-GCATCCTTGTTCATTCTGTGC-3 |
| ***Gastrin*** | 5’-AATGTAGACGGAAGTGGACAGC-3’ | 5’-GCATCCTTGTTCATTCTGTGC-3’ |
| ***Somatostatin*** | 5’-CCCAGACTCCGTCAGTTTCTG-3’ | 5’-GGGCATCATTCTCTGTCTGGTT-3 |
| ***$H^{+}K^{+}$-Atpase*** | 5’-CCGGTGGGTGTGGATCAG-3’ | 5’-GCAAAGAGCCCGGTCATG-3’ |
| ***Rpl32*** | 5’GAGGTGCTGCTGATGTGC-3' | 5’GGCGTTGGGATTGGTGACT-3' |
